# Geometric Diagrams of Genomes: constructing a visual grammar for 3D genomics

**DOI:** 10.1186/s13059-025-03646-y

**Published:** 2025-06-26

**Authors:** Carla Molins-Pitarch, Jonathan Khao, Santiago Bonet, Yolanda Justicia, Clementina Altube, Mike Goodstadt, Francesc Ribot, Erez Lieberman Aiden, Nicola Neretti, Gaël G. McGill, Marc A. Marti-Renom

**Affiliations:** 1 Image Processing and Multimedia Technology Center, Universitat Politècnica de Catalunya-Barcelona Tech, 08222 Terrassa, Spain; 2 Digizyme Inc, Brookline, MA USA; 3 Elisava, Barcelona School of Design and Engineering (UVic-UCC), 08002 Barcelona, Spain; 4 Centre Nacional d’Anàlisi Genòmica (CNAG), Baldiri I Reixac 4, 08028 Barcelona, Spain; 5 Center for Genome Architecture, Department of Molecular and Human Genetics, Baylor College of Medicine, Houston, TX USA; 6 Department of Molecular Biology, Cell Biology and Biochemistry, Brown University, Providence, RI 02912 USA; 7 Center for Molecular and Cellular Dynamics, Department of Biological Chemistry and Molecular Pharmacology, Harvard Medical School, Boston, MA USA; 8 Centre for Genomic Regulation (CRG), Barcelona Institute of Science and Technology (BIST), Dr. Aiguader 88, 08003 Barcelona, Spain; 9 ICREA, Pg. Lluís Companys 23, 08010 Barcelona, Spain

## Abstract

Advances in the field of three-dimensional (3D) genomics have revealed an ever-expanding array of architectural features that were unknown only a few years ago. Just as ribbon diagrams integrate spatial and symbolic representation to communicate the shape of a protein, the representation of genomes in 3D space requires the development and use of new cartographic symbols and visual conventions. Here, we propose a conceptualized grammar that makes it easier to create visual 3D representations of genomes.

**Supplementary Information:**

The online version contains supplementary material available at 10.1186/s13059-025-03646-y.

## Background

The use of symbolic representations of structural features is an essential aspect of cartography—ranging from subway maps to depictions of biomolecules. For instance, as the field of protein folding expanded and increasingly complex protein structures were solved, the need for effective symbolic representations grew. This led to the development of ribbon diagrams by Jane Richardson in the 1980 s [1], which became indispensable in supporting the growing field of structural biology. Indeed, protein structure visualization has remained largely unchanged since the introduction of ribbon diagrams, despite significant advances in structural determination methods and, more recently, AI-based folding predictions [2]. While accuracy measures for protein models exist independently of their visual representation, spatial mapping of these metrics onto 3D structures has been essential for assessing model reliability. Over the decades, this approach has helped the protein structure community contextualize model accuracy and interpret biological relevance.

These same needs are now emerging in the field of 3D genomics, where, thanks to newly developed microscopy, molecular genomics, and computational approaches, the spatial organization of genomes is being explored at unprecedented resolutions [3, 4]. Nowadays, we know that the genome organizes at different scales in the nucleus in a nonrandom fashion. First, chromosomes are organized as territories occupying preferential positions depending on their size [5–7]. Second, chromatin organizes itself into compartments of varied genomic sizes, both within and across chromosomes, that co-localize in space based on their chromatin state [8]. And third, chromatin is further organized into loops [9], stripes, and domains [10, 11]. This last finer scale of organization is thought to favor the establishment of chromatin contacts involved in the regulation of transcription and in the specificity of cell transcriptomes [12]. In summary, characterizing how the genome is folded in the nucleus is essential to determine how it can constrain or regulate the nuclear processing of DNA including transcription, replication, and repair [13, 14].

Like proteins, once a 3D model of a genome is determined (or predicted), it must be visualized to extract maximal biological insights into the molecular mechanisms associated with its folding and function. For example, genome structure has previously been analyzed and represented as a graph, where nodes correspond to genomic loci and edges represent interaction frequencies derived from Hi-C data between these loci [15]. Norton and colleagues applied network modularity optimization to identify hierarchical structures such as genomic domains, capturing the genome’s multi-scale organization [16]. These types of representations are highly useful for understanding how nested domains arise and their relationships. However, they do not provide specific representations for different genomic scales. Moreover, graphs are primarily designed for 2D space, making them difficult to visualize in 3D, where cluttering hinders user interpretation.

Fortunately, genomic data can be processed and visualized graphically for fast and precise communication. Genome browsers have historically used graphical elements—typically displayed as horizontal tracks, aligned and stacked below the reference nucleotide sequence—to communicate complex 1D and 2D genomic data, with the UCSC Genome Browser being a prime example [17]. However, these genomic tracks were neither conceived nor designed to handle explicit 3D data, leading to potential distortion and occlusion due to perspective, lighting, and the complexities of human visual processing [18]. Moreover, representing the genome in 3D presents additional challenges: (i) a proliferation of topological complexity, making side-by-side comparisons very difficult, (ii) the inherent difficulty of representing a 3D object on a fundamentally 2D platform (e.g., a computer screen), and (iii) the challenge of conveying spatial relationships (e.g., proximity) between functionally related genomic elements. In summary, we now face the challenge of defining a visual grammar that best represents 3D genomic data and datasets, which is essential for accurate communication [19].

A Catalan version of the summary of this article is available as supplementary file Additional file 1 as well as at 10.5281/zenodo.15512695. [20].

## Results and discussion

Here, we are proposing the *Geometric Diagrams of Genomes* (GDG), a visual grammar for 3D genomics. GDG builds on the conceptual insights obtained by interpreting nuclear ligation assays [21] such as Chromosome Conformation Capture (3C) [22] and derivatives and/or microscopy-based experiments, namely: the existence of chromosome territories (*circle*), compartments (*square*), domains/stripes (*triangle*), and loops (*line*). As of today, genomes have been visualized with a variety of representations focusing on chromatin strands rather than other higher-order structures (Additional file 2: Fig. S1), which has precluded standardized 3D renderings of genomes. Part of the problem arises from the field heavily relying on the use of generalized 3D browsers for biomolecules, which have been previously developed for representing proteins, RNAs, and their complexes. As such software packages were never developed for rendering genomes in space and time, their visualizations are usually limited in the number of representations ranging from “ball-and-stick” to “worm-like” visualizations. Unfortunately, these visual grammars cannot convey the complexity of spatially representing what is the largest biomolecule of a cell (i.e., the human genome composed by 3 × 10^9^ elements or nucleotides), whose scale spans ~ 4 orders of magnitude (from 0.34 nm for nucleotides to the > 10 um for the nucleus). Besides, color and texture of renderings in 3D genomics have been used arbitrarily with no pre-existing conceptualization. To address these limitations, we next propose a series of principles behind the GDG visual grammar with the hope that they could be adopted by the new breed of specialized 3D genome browsers.

### Genome structural elements

**Fig. 1:**
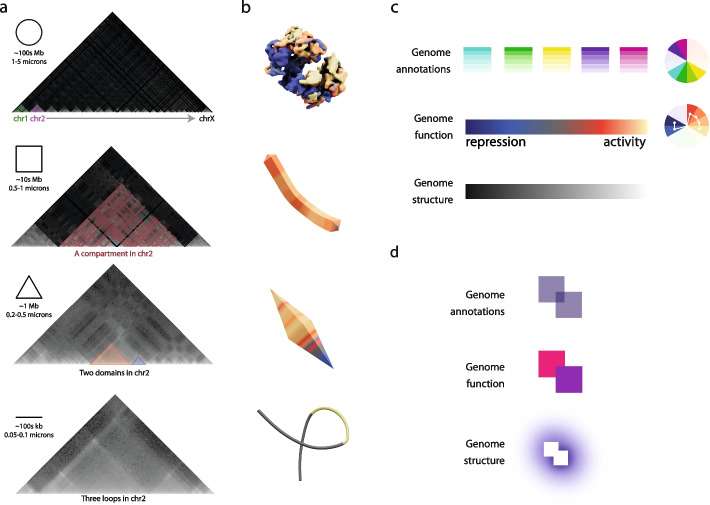
Higher-order hierarchical structural elements of the genome.** a** Top to bottom: *Circle* level (genome scale). Hi-C interaction maps confirmed the existence of chromosome territories previously discovered by microscopy [7]. *Square* level (Mb scale). At the chromosome level, Hi-C interaction maps revealed the existence of genomic compartments [8]. *Triangle* level (hundreds of kb scale). Similarly, Hi-C interaction maps clearly revealed strong signal along the diagonal called topologically associating domains or TADs [10, 11]. *Line* level (tens of kb). Finally, loops were also revealed by analyzing high-resolution Hi-C interaction maps [9]. **b** *Form*. *Circle* level (chromosome scale), *Square* level (compartment scale), *Triangle* level (domain scale), and *Loop* level (gene scale). Examples on the use of each of the color, texture, and form in each level of resolution are shown. **c** *Color.* Bright colors to genome annotations (i.e., genes, transcripts) and cold (blue) to hot (red/yellow) colors for genome function indicating activity (*i*.e., ChIP-seq, RNA-seq).** d** *Texture.* Transparency for to genome annotations, solid for genome function and glowing for highlighting 3D aspects that otherwise can be occluded by the intrinsic depth in the screen

Thanks to light microscopy and specially Hi-C experiments [8], we now know that the genome non-randomly organizes at several 3D scales (Fig. 1a, b). First, at the highest scale of organization (lowest resolution), the genome is organized into chromosome territories, which occupy a discrete space within the nucleus [7]. As chromosomes are formed by a very large polymer of chromatin (in higher eukaryotes up to hundreds of Megabases of DNA), their unpacking after mitosis precludes them from fully exploring the entire confined space of the nucleus. Such physical constraint results in each individual chromosome occupying during interphase a limited space that can be roughly approached by a *spheroid* with diameters of the order of one to few micrometers [23]. Next, at the scale of few megabases (Mb), the second level of organization of genomes is the so-called compartments [8, 9, 24]. Genome compartments were discovered by analyzing Hi-C maps and were defined as genome regions of varied sizes that co-localize in space based on their chromatin state. In Hi-C maps, compartments visually resemble *squares* or *rectangles*. Third, compartments were further sub-divided into domains, which are self-interacting regions of the genome that appear as visual *triangles* at diagonal in Hi-C chromatin interaction maps. Domains, also called topologically associating domains or TADs, although accepted by the community, have been the center of strong debates on their definition, biological relevance, and even their existence [25]. However, one accepted feature of domains is their intrinsic capacity to physically modulate the probability of formation of loops, the finest level of structural organization in the genome. At the tens of kilobases (kb) scale, chromatin forms physical loops bringing DNA regions in close proximity, which is facilitated by a loop extrusion process involving several protein complexes including, among others, CTCF and cohesin [26–28]. Such loops have classically been drawn as a continuous solid *line* folded onto itself.

In summary, the GDG visual grammar builds on a set of geometrical shapes of circles, squares, triangles, and lines to propose specific forms for representing in 3D chromosomes, compartments, domains and loops, respectively. Based on this set of shapes, each scale will correspond to a geometrical form in a tri-dimensional space according to its bi-dimensional instance. The goal for using such simple geometric forms is to provide the viewer an object that visually relates to accepted concepts on higher-order genome organization while providing attributes to each form according to its scale.

As data visualization requires its mapping to color and form to ensure keeping the essence of the data visual to a proper perception [29], a new visual grammar needs to encompass the rules of the visual language in terms of form, color, and texture. Next, we outline one possible use of form, color, and texture to represent genomes using the GDG visual grammar. Many of the aspects that we will introduce in the next sections were thought considering how genomes are currently represented in linear or bidimensional tracks such as 1D tracks for gene annotation or 2D tracks for gene expression, respectively. However, these are just a proposal with the hope that such rules will enable people to broadly approach the challenge of visualizing genomes in 3D in a similar way in which Ribbon Diagrams provided the basis on how we visualize proteins today [1].

### Form, color and texture

#### ***Form (***Fig. 1b*** and “***Methods***”)***

As indicated above, the GDG visual grammar follows the scales of the genome in 3D and their visual representations as *Circle* level (chromosome scale), *Square* level (compartment scale) *Triangle* level (domain scale), and *Line* level (loop scale). These shapes are translated into 3D forms as globular pseudo-spheres, irregular prisms, bipyramids, and tubular representations for chromosomes, compartments, domains, and loops, respectively. Such representation will allow users to share biological concepts such as compartments, domains, and loops in an intuitive manner similarly to helices and betas in protein representation.

#### ***Color (***Fig. 1c*** and “***Methods***”)***

Color is the graphical variable [30] which value, hue, and intensity together with color theory, prompted us to create different, contrasted, and compatible color palettes for sequence annotations (non-sequential colors), genome activity (continuous color scale), and 3D structure (black to white gradient). Starting with a trichromatic color wheel based on the three primary colors and three secondary colors, we produced a 16-color wheel for annotation of genome features into the 3D models of the genome. The annotation color wheel was produced by adjusting the base colors hue to get high-contrasting tones and excluding shades traditionally related to function (dark blues and reds) to prevent confusion. The aim of these hues and its adjustable intensities (due to the translucency offered by the texture) is to create enough contrast and intelligibility when stacking and overlapping colors on genome annotations. The second color wheel defines two ranges for a continuous color scale for function. This continuous selection of cold-to-hot colors for annotating functional activity of the genome (*i.e.,* blue for non-active and red-yellow for active genome) opposes high brightness to muted colors while having a neutral middle point with grey tones. Visually both ends of the color scale are opposed in brightness while offering a seamless but contrasted transition from end to middle and middle to end. Finally, a black-to-white scale is reserved specifically to indicate spatial proximity of elements in 3D being white closest point to viewer and black further point. This final scale allows for a high contrast and simplicity, which is needed in a cluttered 3D space, and at the same time provides depth perception by just applying a monochrome shading.

#### ***Texture (***Fig. 1d*** and “***Methods***”)***

By default, textures in 3D representations are plain colors without considering lighting and shadow casting. GDG proposes the use of at least three types of texture renderings to be applied on the forms for representing genomes in 3D. First, a solid (100% opacity) texture is reserved for specific genome functional annotations at the compartment or chromosome levels. A solid texture will not allow for visual overlap, which might allow for a clear distinction of elements. Second, a transparent (< 100% opacity) texture is reserved to highlight genome annotations at the high-resolution level (loop level) as annotation can often overlap in sequence coordinates. When adjusting the transparency of the texture, the color of the annotation will become translucent, creating a new level of information when there is an overlap with other annotations facilitating the identification of the beginning and end of a particular annotation. Third, a glowing (emissive) texture can be used to specifically highlight certain aspects of the genome in 3D that otherwise would be hidden or cluttered by the intrinsic 3D occlusion problem. Such glowing texture is visible to the user even when occluded, in a similar way a light can be seen open behind a door. However, such glowing texture would need to be reserved to a minimal number of distinctive elements to be effective.

### Transitions between scales

The GDG visual grammar proposes different genomic scales corresponding to the levels of genome organization in space and time. A proper transition between the different states will aid the user in navigating the scales. GDG proposes 3D renderings with smooth morphing transitions between scales (Additional files 3 and 4: Videos 1 and 2). Transitions are accomplished when the canvas that the user is presented with is focused on the amount of DNA that transitions to the next upper or lower level of organization.

### GDG and 3D genome browsers

Genome browsers have been developed for decades now at the trail of great advances in genomics. However, such genome browsers only very recently are explicitly making use of 3D models of genomes rendered in the Cartesian space. Prime examples of such browsers include SpaceWalk http://aidenlab.org/spacewalk or the Nucleome Browser http://vis.nucleome.org, among others. One of the common characteristics of such browsers is the capacity of the user interface to connect via simple interactions both the classical genome tracks and the newer Hi-C interaction maps as well as the genome 3D coordinates. However, a rapid visit to the existing browsers clearly shows the diverse rendering of the genome in 3D and the lack of specialized renderings depending on the scale of the visualization. In fact, one needs to know explicitly the amount of DNA on the screen to assess the scale of the object that the user is visualizing. In other words, it is currently impossible to know if the user is visualizing a domain, a compartment, or a local loop unless explicitly indicated. We trust that if such 3D genome browsers adopt the GDG rules, the initial visual assessment of the genome structure would contain implicitly this information among many others. If 3D genome browsers follow the same guidelines for displaying data, it would allow researchers to directly compare images generated by different browsers (including the ones published in research articles), just like how we can easily compare protein or RNA structures visualized using common principles by different software.

### GDG insights into biology

The adoption of new visualization techniques in 3D genomics can significantly aid experimentalists in designing more informed and targeted experiments. The GDG grammar provides an intuitive and comprehensive view of chromatin organization, highlighting structural features such as loops, TADs, compartments, and chromosomes with greater clarity. Such representations, when integrated with multi-omics data, can pinpoint key regulatory interactions, identify potential enhancer–promoter contacts, and detect structural variations that may influence gene expression. This enhanced spatial perspective could aid experimentalists to refine their hypotheses, select optimal regions for perturbation, and design experiments that more precisely test the functional consequences of genome architecture, ultimately improving the efficiency and accuracy of their investigations.

**Fig. 2:**
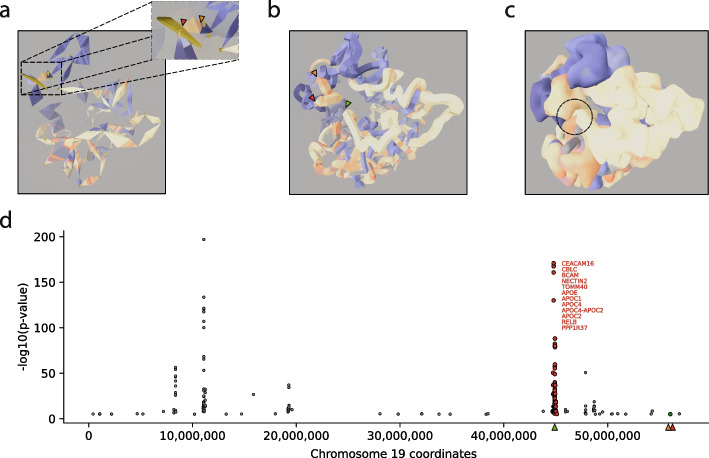
Example of biological insights from GDG grammar.** a** *Triangle* level representation (TADs) of a chromosome 19 model for IMR90 cells (Methods). The inner zoom indicates the presence of a small (390 kb) TAD within a stretch of larger TADs at the end of the chromosome (chr19:55,860,000–56,250,000). This TAD attracts visual attention for two main reasons: first, the TAD is active (yellow/red shades) in the middle of a stretch of non-active TADs (blue shades) and second, its two boundaries (orange and red arrows), specially the up-stream boundary (red arrow), are very strong. **b** *Square* level (compartments) representation. The selected TAD also matches a small and very active compartment whose boundaries (red and orange arrows) delimit very inactive compartment stretches. Interestingly, such compartment is spatially close to another region in the chromosome (green arrow) that is 11.6 Mb away of the active TAD represented in panel **a** (chr19:44,610,000–45,180,000; 570 kb). **c** *Circle* level (chromosomes) representation. The chromosome is clearly partitioned into two compartments (active in yellow–red and inactive in blue). Of particular interest is the region highlighted with a dashed circle showing the spatial proximity of sequentially separated active compartments, one of which stands out from the inactive compartment. **d**. SNPs associated to “*lipid measurement*” and residing in the human chromosome 19. Each dot represents a SNP. The green dot represents an isolated orphan SNP residing in the selected small active TAD within a largely inactive part of chromosome 19. Red dots indicate SNPs in the selected interacting region with the TAD, which contain several SNPs and genes associated to lipid metabolism. The color arrows in the x-axis correspond to the arrows in the 3D representations in panels **a** and **b**

To assess whether the new GDG visual grammar helps define biological hypotheses, we built a 3D model of the human chromosome 19 from Hi-C maps of IMR90 cells [9] using TADbit (Fig. 2 and “ Methods”). The model was next visualized at all scales using the GDG visual grammar, which allowed us to quickly discover a TAD of interest located at chr19:55,860,000–56,250,000. This TAD, a relatively small for the human genome (390 kb), was selected because it is an active TAD in the middle of a stretch of inactive chromatin (Fig. 2a). The identified TAD harbors the Galectin-associated protein (GALP gene), three NOD-like receptor family pyrin domain-containing genes (NLRP13, NLRP8 and NLRP5), and five zinc finger genes (ZNF787, ZNF444, ZSCAN5B and ZSCAN5C) known to bind to DNA and regulate gene expression. Interestingly, a search for Single Nucleotide Polymorphisms (SNPs) within this selected TAD in the GWAS Catalog indicates that there are a total of 37 traits associated with this region. One of those traits with orphan gene assignments is “*lipid measurement*” with no gene nearby in sequence that can be clearly associated with lipid metabolism. Additionally, the GWAS Catalog includes 230 SNPs across chromosome 19 associated with “*lipid measurement*” trait, which are particularly enriched in two genomic locations (Fig. 2d). The first genomic region at the beginning of chromosome 19 (~ 10.1 Mb) is spatially far from the selected TAD region. However, the second region around Mb 44.6 is enriched in SNPs associated with lipid metabolism as well as genes of the APO family of apolipoproteins that play critical roles in maintaining healthy lipid levels. This region is in spatial proximity as well as sharing an active compartment with the selected TAD (Fig. 2b–c). Altogether, the model represented using GDG visual grammar helps build the hypothesis that a variant located more than 11.6 Mb away from a particular region of the genome could be associated with a cluster of genes related to lipid metabolism. We envision that hypotheses could be generated more rapidly by using the GDG visual grammar when representing genomes in space and time. It is important to note that, when models are built using only partial data (e.g., a single chromosome in Fig. 2), they may not fully reflect the true complete spatial organization of the genome, as interactions with other chromosomes are absent. Therefore, in the example provided, one could be missing additional biologically relevant interactions with other parts of the genome. However, the fundamental principles of its representation proposed by the GDG grammar remain unchanged as the visualization framework is independent of the specific modeling approach used or the modeled region. Thus, whether a model is applied to an entire genome or a specific genomic region, the graphical representation follows the same conventions. This distinction is crucial to understanding that the method of representation is designed to be consistent, regardless of the genomic scope of the underlying model.

### Challenges

The 3D genomics or 4D nucleome field is relatively new; many of the concepts and principles of genome folding have been discovered and formalized recently, and some of them are still in active debate. Moreover, standardization of the methods and data has only recently been addressed. Therefore, the GDG visual grammar proposed here may likely be continuously challenged in the years to come. We can now envision a few of those challenges.

First, do all 3D genome scales and levels apply equally to all organisms? We already know that many of the aspects discussed here apply to most eukaryotic cells with certain specificities and that many prokaryotic cells also contain most levels of organization including the domains or CIDs. However, not all species may contain all levels of organization nor the size of the different levels in terms of DNA content will be constant between species. Therefore, the scales of the renderings will need to be adapted to the studied specie.

Second, is the genome a static 3D object or a dynamic one? As observed by imaging technologies, genomes or genomic domains can adopt very different conformations at all scales when compared between individual cells [31–33]. While some structural features, such as compartments and territories, are now recognized as being present in single cells, others, particularly TADs and loops, appear less distinct at the single-cell level compared to population-based data. As single-cell chromosome conformation capture and imaging techniques continue to advance, our understanding of these structures will improve. However, since the focus of the GDG is not on defining these features but on their visual representation, its applicability to single-cell models could be adapted, especially where structural boundaries between TADs, for example, are less well-defined or blurred. Additionally, representing variability in 3D has always been a challenge and this is not an exception for 3D genomics [34] where structural variability is now for the first time being classified [35]. Such structural variability in biological models arises from many sources including uncertainty in the modeling process, experimental artifacts, and real biological variability. In the protein field, such variability is often addressed through ensemble representations, where multiple conformations are depicted simultaneously, typically as overlaid structures, smoothed confidence intervals, or color-coded uncertainty scores. Methods such as ensemble NMR structures, molecular dynamics simulations, and integrative modeling provide frameworks for visualizing these variations. Additionally, tools like B-factors in X-ray crystallography and per-residue confidence scores enable the direct representation of uncertainty in static visualizations. In contrast, genome structure visualization presents additional challenges due to the scale and complexity of chromatin folding. While protein models typically involve well-defined atomic coordinates, genome models depend on probabilistic reconstructions, often lacking single-molecule resolution. To visually convey variability in genome structures, we propose using ensemble-based visual representations, akin to those in protein modeling. For example, overlapping multiple genome conformations in semi-transparent rendering can reflect model uncertainty. Similarly, color gradients or thickness variations along chromatin paths could indicate structural confidence, where highly uncertain regions fade or blur. Additionally, animation-based representations could illustrate the dynamic range of possible genome conformations, conveying both static variability and dynamic fluctuations.

Finally, do we already know all the scales of genome folding? As the 3D genomics field advances, new technologies will be developed and existing ones will be further explored, for example in the recently developed Ultra-Hi-C maps [24]. Such advances will likely bring new insights on the first principles that govern genome folding at the currently explored scales, which will be further linked to existing higher-resolution genome scales (i.e., the nucleosome). Unequivocally, the new insights will bring new levels of genome organization that are currently not considered by the GDG visual grammar. For example, stripes (also termed “flares”) and fountains (also called “jets”) are emerging and, if validated for their functional relevance, will be incorporated into future revisions of the GDG visual grammar. Despite the ongoing debate surrounding the definition and acceptance of some of these structural features, we argue that the biological relevance of each of those elements can only be fully explored if they are effectively visualized and standardized, facilitating data interpretation and knowledge dissemination across the field. Nevertheless, a continuous adaptation of the grammar will invariably be needed.

## Conclusions

The proposed GDG visual grammar provides a framework towards a unified representation of genomes in space, which could allow for easy interpretable exchange of 3D models. A unified grammar has many advantages to the expanding 4D Nucleome community and, to fully benefit from this, we encourage software developers to adopt the rules proposed by the GDG visual grammar in future developments of existing and new 3D genome browsers. As happened in the field of proteins 40 years ago [1], the 3D genomics/4D nucleome field is in a mature stage that, with the help of standardized visualizations, will bring new insights into all dimensions of the genome.

## Methods

### 3D modeling

To ensure the applicability of this newly developed visual grammar to experimentally derived data, we applied GDG to a model of the human chromosome 19 from IMR90 cells, which had a Hi-C dataset previously published [9]. Models were built at 30 kb resolution using the TADbit modeling tool [36] with default parameters. Compartments and TADs were measured with TADbit using default parameters as well.

The following features derived from the Hi-C data were included in the visual grammar (Additional file 5: Data file 1):

- XYZ coordinates.
- Gene boundaries for hg38 annotation.
- TAD insulation score, identified boundaries, and their computed insulation strength.
- A/B compartments, along with their eigenvalues.

The XYZ coordinates were used to instantiate points in 3D space, where the remaining listed features were stored as point metadata. Centromeric and telomeric regions whose Hi-C data was missing from the dataset were omitted from the visualized model.

### Prototyping

The general 3D modelling software Autodesk Maya [37], Molecular Maya [38], Maya’s Bifrost Editor visual programming environment [39], and additional Bifrost open-source libraries [40] were chosen for their versatility and fast prototyping capabilities during initial explorations and subsequent iterative refinement of the visual language. The prototyping includes all the described levels of genome organization and is available at https://tinyurl.com/GDGGrammar. Briefly, the levels are defined as:

1. Loop (line) level. The XYZ coordinates of the genomic bins were sequentially connected to illustrate the chromosome at the loop level, where loop segments inherit from the metadata of the starting genomic bin’s metadata. Given the inherent noise of the XYZ dataset, centripetal Catmull-Rom splines were used to generate smooth curves to provide a continuous visual flow and reduce the visual noise of the loop representation. In this case study, we chose to illustrate genes through color coding, where a loop segment connecting two bins inherits the gene color from the starting bin. Given the 30kbp resolution of the genomic bins, a single bin can include multiple genes. We therefore chose to keep the longest spanning one.
2. Domain (triangle) level. Each domain is represented as a triangular bipyramid, connecting two XYZ points identified TAD boundaries, where the volume of the geometry is proportional to the amount of DNA between boundaries. For long-spanning TADs with boundaries close in space, the volume was clamped to a maximum value to avoid excessively short and thick bi-pyramids. It is important to note that bipyramids use the midpoint as the junction where edges from one pyramid converge with the other. This could affect how the data is interpreted. For example, if a particular TAD in the linear representation results in most of the DNA being modeled at the end of the TAD rather than the center, one might interpret a homogeneous distribution of DNA between the TAD boundaries. Finally, the geometry was then divided along its length by its number of bins, where each subdivision was coloured following the corresponding bin’s compartment eigenvalue. Flat triangles were illustrated at TAD boundaries, with a size proportional to the boundary’s insulation score.
3. Compartment (square) level. Based on the loop representation previously described, loop segments were extracted between compartment boundaries and smoothed by iteratively averaging neighboring point positions, while preserving the start and end point positions. Prisms following the smoothed line were then generated, with a volume proportional to the amount of DNA in each compartment segment. Similarly, at the domain level representation, the geometry was subdivided along its length, and each subdivision was colored following the bin’s compartment eigenvalue.
4. Chromosome (sphere) level. Based on the loop level representation, control points from the previously generated Catmull-Rom spline were extracted, and a point-meshing algorithm was used to generate a surface mesh (using Bifrost Editor’s native point meshing tools) colored according to the bin’s compartment eigenvalue. The size of the meshed points and the meshing threshold were adjusted to provide a smooth surface that englobes the compartment level representation.

### Eigenvalue scale bar

To provide more visual contrast for the scale bar, values between the 5th and 95th percentiles were kept. The positive and negative eigenvalues were independently normalized to symmetrize the color scale bar.

## Supplementary Information

Additional file 1: A Catalan translation of the summary of this article

Additional file 2: Supplementary Fig. S1. Diverse representations of 3D genomes and genomic domains. Images are ordered from “ball-and-stick” to “worm-like” renderings, which showcase the large variability of forms, colors and textures used to represent genomes in 3D

Additional file 3: Supplementary Video 1. Supplementary_Video_1_GDG.mov. Short explanation of the GDG grammar for visualizing genomes and genomic domains in 3D

Additional file 4: Supplementary Video 2. Supplementary_Video_2_GDG_explanation.mov. Detailed explanation of the GDG grammar for visualizing genomes and genomic domains in 3D

Additional file 5: Supplementary Data 1. Supplementary_Data_GDG.zip. Zip data file containing the example model of human chromosome 19, compartment assignments, TAD border definitions, and gene coordinates

## Data Availability

Data availability The Hi-C dataset for IMR90 [9] used to model chromosome 19 structure was downloaded from ENCODE experiment ENCSR852KQC [41].

## Code availability

The code for modeling 3D genomes from Hi-C data used in this article is available at https://github.com/3DGenomes/TADbit [36].
