# Supplementary material for: Geometric Diagrams of Genomes: constructing a visual grammar for 3D genomics
Source: Genome Biol. 2025 Jun 26;26:181. doi: 10.1186/s13059-025-03646-y (PMC12203721; doi:10.1186/s13059-025-03646-y)
Supplement: Supplementary file 1 — Additional file 1: A Catalan translation of the summary of this article [file 13059_2025_3646_MOESM1_ESM.pdf]

Disclaimer: This translation in Catalan was submitted by the authors and we reproduce it as supplied. It has not been peer reviewed. Our editorial processes have only been applied to the original abstract in English, which should serve as reference for this article. This translated abstract is published under the same licence as the article.

Diagrames geomètrics de genomes: construcció d'una gramàtica visual per a la genòmica 3D

Carla Molins-Pitarch[1], Jonathan Khao[2], Santiago Bonet[3], Yolanda Justicia[3], Clementina Altube[3], Mike Goodstadt[4], Francesc Ribot[3], Erez Lieberman Aiden[5], Nicola Neretti[6], Gaël G. McGill[2,7], and Marc A. Marti-Renom[4,8,9,*]

1. Image Processing and Multimedia Technology Center, Universitat Politècnica de Catalunya-Barcelona Tech, 08222 Terrassa, Spain2. Digizyme Inc., Brookline, MA, USA
2. Elisava, Barcelona School of Design and Engineering (UVic-UCC), 08002 Barcelona, Spain
3. Centre Nacional d'Anàlisi Genòmica (CNAG), Baldiri i Reixac 4, 08028 Barcelona, Spain
4. Center for Genome Architecture, Department of Molecular and Human Genetics, Baylor College of Medicine, Houston, Texas, USA
5. Department of Molecular Biology, Cell Biology and Biochemistry, Brown University, Providence, RI 02912, USA
6. Center for Molecular and Cellular Dynamics, Department of Biological Chemistry and Molecular Pharmacology, Harvard Medical School, Boston, MA, USA
7. Centre for Genomic Regulation (CRG), Barcelona Institute of Science and Technology (BIST), Dr. Aiguader 88, 08003 Barcelona, Spain
8. ICREA, Pg. Lluís Companys 23, 08010 Barcelona, Spain

* La correspondència en català sobre aquest article es pot adreçar a: martirenom@cnag.eu

Resum

Els avenços recents en la genòmica tridimensional (3D) han revelat una arquitectura nuclear altament organitzada i jeràrquica que va més enllà de la seqüencia lineal del genoma. De manera similar a com els diagrames de cintes van ajudar a la representació d'estructures de les proteïnes, es fa necessari establir una gramàtica visual que permeti representar la conformació tridimensional del genoma de manera estandarditzada, intuïtiva i informativa.

Aquí es proposa la gramàtica visual "Diagrames Geomètrics de Genomes" (*Geometric Diagrams of Genomes* o GDG), una visualització que associa formes geomètriques simples amb les diferents escales d'organització del genoma: cercle per a territoris cromosòmics, quadrat per a compartiments, triangle per a dominis i línia per als llaços. Aquesta codificació formal permet traslladar informació estructural a un entorn 3D de forma clara i sistemàtica.

A escala de representació gràfica, la gramàtica GDG defineix regles per a l'ús de la forma, el color i la textura. La forma expressa l'escala de l'organització. El color utilitza rodes cromàtiques diferenciades per anotacions genòmiques, activitat funcional (blau-vermell-groc) i proximitat

espacial (escala blanc-negre). Les textures (opacitat, transparència, emissivitat) afegeixen capes d'informació, especialment útils en entorns 3D on l'oclusió visual pot ser un problema.

La proposta del GDG contempla també els reptes emergents en la genòmica 3D: Variabilitat entre cèl·lules, naturalesa dinàmica de les estructures, reptes de normalització i estandardització visual, i la possibilitat de noves escales com franges (*stripes*) o rajos (*jets*) no considerades encara per GDG.

En conclusió, el GDG s'estableix com un llenguatge visual estandarditzat per la representació del genoma en espai, anàleg als diagrames de cintes en biologia estructural de proteïnes. La seva adopció podria millorar la interpretació visual, facilitar la comparació entre navegadors genòmics 3D, i accelerar la formulació d'hipòtesis experimentals en el context de la genòmica estructural.
