# Supplementary material for: Geometric Diagrams of Genomes: constructing a visual grammar for 3D genomics
Source: Genome Biol. 2025 Jun 26;26:181. doi: 10.1186/s13059-025-03646-y (PMC12203721; doi:10.1186/s13059-025-03646-y)
Supplement: Supplementary file 2 — Additional file 2: Supplementary Fig. S1. Diverse representations of 3D genomes and genomic domains. Images are ordered from “ball-and-stick” to “worm-like” renderings, which showcase the large variability of forms, colors and textures used to represent genomes in 3D [file 13059_2025_3646_MOESM2_ESM.docx]

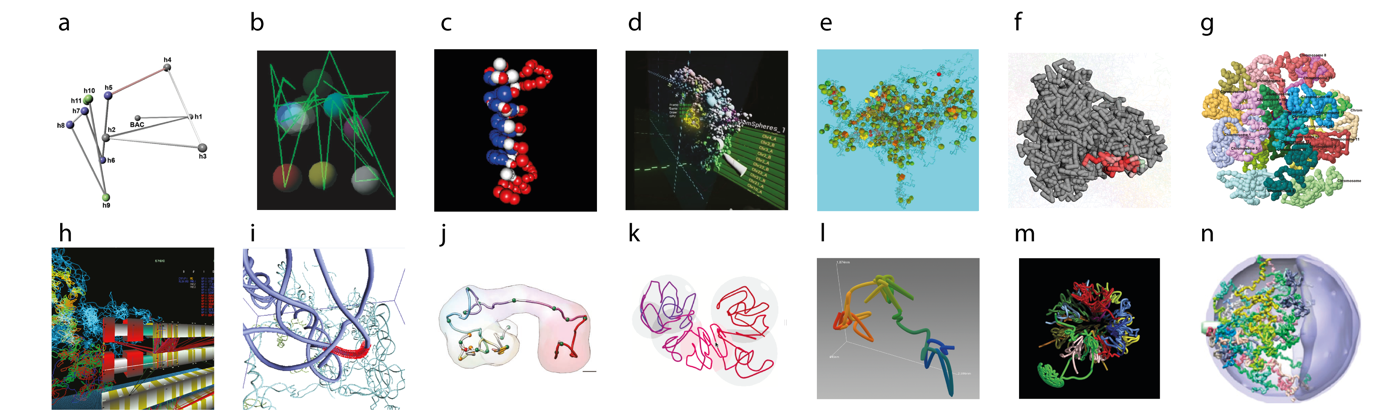


**Fig. S1. Diverse representations of 3D genomes and genomic domains.** Images are ordered from “ball-and-stick” to “worm-like” renderings, which showcase the large variability of forms, colors and textures used to represent genomes in 3D. For example, balls (spheres) have been used at several different scales from sub-domain (panels a, b, and j), domains (panel c), or chromosome (panel d). Color has been used in almost any context to delineate objects, functions, selection, sequence order/localization, etc. Finally, texture has been used to arbitrarily render diverse objects such nucleosomes (panel e), genes (panel i), chromatin states (panel h), chromosomes (panel d) or nuclear bodies (panel n). All panel images were obtained from published works or existing rendering packages: **a** Jhunjhunwala, et al. (2008)[1]; **b** Fraser, et al. (2009)[2]; **c** Hu, et al. (2013)[3]; **d** Chrom3D-VR; **e** Genome3D[4]; **f** Nucleome Browser (<http://vis.nucleome.org>); **g** Gmol[5]; **h** Globe3DV; **i** 3DGB[6]; **j** Baù, et al. (2011)[7]; **k** TADkit (<https://github.com/3DGenomes/TADkit>); **l** SpaceWalk (<https://github.com/igvteam/spacewalk>); **m** Duan, et al. (2010)[8]; **n** Tjong, et al. (2012)[9];

**References**

1. Jhunjhunwala S, van Zelm MC, Peak MM, Cutchin S, Riblet R, van Dongen JJ, Grosveld FG, Knoch TA, Murre C: **The 3D structure of the immunoglobulin heavy-chain locus: implications for long-range genomic interactions.** *Cell* 2008, **133:**265-279.

2. Fraser J, Rousseau M, Shenker S, Ferraiuolo MA, Hayashizaki Y, Blanchette M, Dostie J: **Chromatin conformation signatures of cellular differentiation.** *Genome Biol* 2009, **10:**R37.

3. Hu M, Deng K, Qin Z, Dixon J, Selvaraj S, Fang J, Ren B, Liu JS: **Bayesian inference of spatial organizations of chromosomes.** *PLoS Comput Biol* 2013, **9:**e1002893.

4. Asbury TM, Mitman M, Tang J, Zheng WJ: **Genome3D: a viewer-model framework for integrating and visualizing multi-scale epigenomic information within a three-dimensional genome.** *BMC Bioinformatics* 2010, **11:**444.

5. Nowotny J, Wells A, Oluwadare O, Xu L, Cao R, Trieu T, He C, Cheng J: **GMOL: An Interactive Tool for 3D Genome Structure Visualization.** *Sci Rep* 2016, **6:**20802.

6. Butyaev A, Mavlyutov R, Blanchette M, Cudre-Mauroux P, Waldispuhl J: **A low-latency, big database system and browser for storage, querying and visualization of 3D genomic data.** *Nucleic Acids Res* 2015, **43:**e103.

7. Baù D, Sanyal A, Lajoie BR, Capriotti E, Byron M, Lawrence JB, Dekker J, Marti-Renom MA: **The three-dimensional folding of the alpha-globin gene domain reveals formation of chromatin globules.** *Nat Struct Mol Biol* 2011, **18:**107-114.

8. Duan Z, Andronescu M, Schutz K, McIlwain S, Kim YJ, Lee C, Shendure J, Fields S, Blau CA, Noble WS: **A three-dimensional model of the yeast genome.** *Nature* 2010, **465:**363.

9. Tjong H, Gong K, Chen L, Alber F: **Physical tethering and volume exclusion determine higher-order genome organization in budding yeast.** *Genome Res* 2012, **22:**1295-1305.
